# Microbes' role in environmental pollution and remediation: a bioeconomy focus approach

**DOI:** 10.3934/microbiol.2024033

**Published:** 2024-08-23

**Authors:** Giuseppe Maglione, Paola Zinno, Alessia Tropea, Cassamo U. Mussagy, Laurent Dufossé, Daniele Giuffrida, Alice Mondello

**Affiliations:** 1 Institute for the Animal Production System in the Mediterranean Environment (ISPAAM), National Research Council, Piazzale Enrico Fermi 1, 80055 Portici, Italy; 2 Messina Institute of Technology c/o Department of Chemical, Biological, Pharmaceutical and Environmental Sciences, former Veterinary School, University of Messina, Viale G. Palatucci snc 98168–Messina, Italy; 3 Escuela de Agronomía, Facultad de Ciencias Agronómicas y de los Alimentos, Pontificia Universidad Católica de Valparaíso, Quillota 2260000, Chile; 4 CHEMBIOPRO Laboratoire de Chimie et Biotechnologie des Produits Naturels, ESIROI Agroalimentaire, Université de La Réunion, 15 Avenue René Cassin, F-97400 Saint-Denis, Ile de La Réunion, France; 5 Department of Biomedical, Dental, Morphological and Functional Imaging Sciences, University of Messina, Via Consolare Valeria, 98125 Messina, Italy; 6 Department of Economics, University of Messina, Via dei Verdi, 75, 98122 Messina, Italy

**Keywords:** bioremediation, bioeconomy, agro-industrial waste, biorefinery, value-added products

## Abstract

Bioremediation stands as a promising solution amid the escalating challenges posed by environmental pollution. Over the past 25 years, the influx of synthetic chemicals and hazardous contaminants into ecosystems has required innovative approaches for mitigation and restoration. The resilience of these compounds stems from their non-natural existence, distressing both human and environmental health. Microbes take center stage in this scenario, demonstrating their ability of biodegradation to catalyze environmental remediation. Currently, the scientific community supports a straight connection between biorefinery and bioremediation concepts to encourage circular bio/economy practices. This review aimed to give a pre-overview of the state of the art regarding the main microorganisms employed in bioremediation processes and the different bioremediation approaches applied. Moreover, focus has been given to the implementation of bioremediation as a novel approach to agro-industrial waste management, highlighting how it is possible to reduce environmental pollution while still obtaining value-added products with commercial value, meeting the goals of a circular bioeconomy. The main drawbacks and challenges regarding the feasibility of bioremediation were also reported.

## 1. Introduction

Rapid industrialization, urbanization, and constant population growth are threatening the environment because of the consequent increase in pollution levels, carbon footprint, and domestic and agricultural waste generation. Humans' actions have been directly involved in pollution, ecological degradation, climate change, and other issues [1]. Environmental pollution is directly threatening the health and survival of all life forms and disturbing the ecological balance of the planet [2].

To mitigate this global issue, many physical, chemical, and biological approaches have been implemented so far [2],[3]. While physicochemical methods have shown several disadvantages, such as sludge formation and disposal, low efficiency, and high treatment cost, as well as the potential to contribute to environmental pollution, biological treatments, particularly bioremediation processes, are currently gaining more and more interest from both industrial and scientific perspectives [4].

Bioremediation is a process through which environmental pollutant concentration is reduced to a harmless value by applying biological mechanisms primarily carried out by specific wild-type or engineered microorganisms, to produce energy and biomass. Bacteria, fungi, and microalgae are widely distributed in the biosphere and are referred to as the main bio-remediators, due to their fast replication rate and ability to grow in a wide range of environmental conditions. These organisms can be employed as a single organism or in a consortium, being able to restore the original natural environment, preventing future pollution, and modulating the breakdown and conversion of toxins through enzymatic skills [5]–[7].

Bioremediation techniques, mainly consisting of degradation, detoxification, mineralization, or transformation, depend on the type of pollutants (heavy metals, agrochemicals, dyes, hydrocarbons, plastics, greenhouse gases, sewage, wastewater, or agro-industrial waste) and can be applied ex situ and in situ [8],[9]. Moreover, due to the straight relationship between bioremediation and microbial activities, process efficiency can be affected by the same parameters that affect microbial growth, such as aerobic or anaerobic conditions, nutrient concentrations, temperature, pH, and other abiotic factors [9],[10]. Thanks to a multidisciplinary approach of molecular biology and genetic engineering techniques, genetically modified organisms can be obtained and employed to increase their bioremediation microbial ability, in order to reduce the environment's toxic compounds. Thus, the implementation of these techniques will address economic and social benefits, reducing disease risks and waste management costs at the same time, for the achievement of ecological stability and a greener environment [11].

Currently, the scientific community supports the straight connection between biorefinery and bioremediation concepts to encourage circular bio/economy practices by enabling the production of value-added-products carried out by microbial pathways addressed to “zero waste”, in comparison to classical waste management strategies [12],[13].

Microbial biorefinery-based technologies could provide major breakthroughs in bio-based economies through the production of several market-based products such as biopolymers, biofuels, biochemicals, bio-additives, pigments, single-cell proteins, and single-cell oils, through “cascade” and/or “one-pot” fermentation approaches, according with the third type of bioeconomy proposed by Vivien et al. [14], who consider it a biomass-based economy. Suitable economic and scientific strategies in a multidisciplinary approach can support the development of sustainable biorefinery processes by addressing circular bioeconomy goals and bridging the gap between waste/pollution remediation and product recovery [15],[16].

This review aims to give a pre-overview on the state of the art regarding the main microorganisms employed in bioremediation processes applied for the most common causes of environmental pollution, highlighting the different approaches and the affecting factors, as well as reporting the main drawbacks and challenges. Moreover, a focus is given on the implementation of bioremediation as a novel approach to agro-industrial waste management. In this regard, the main techniques applied for reducing the negative environmental effects of agro-industrial waste are described, highlighting the importance of carrying out this kind of remediation in order to reduce environmental pollution while still obtaining value-added products with commercial value, meeting the goals of a circular bioeconomy.

## 2. Microorganisms involved in bioremediation

In recent years, bioremediation has garnered significant scientific interest, with mechanisms such as redox reactions, absorption, and alterations in the medium's properties playing crucial roles in the process. Microbial techniques for eliminating heavy metals [17]–[19], including biosorption-bioaccumulation, oxidation-reduction, and biosurfactant production, have gained prominence. In this context, microorganisms are not only employed for heavy metal bioremediation but also for the removal of plastics, dyes, and petroleum-based contaminants [20]–[23].

As previously mentioned, microorganisms play a crucial role in heavy metal pollution reduction by absorbing these metals either actively (bioaccumulation) or passively (adsorption). For instance, lead, mercury, and nickel can be bioremediated by *Saccharomyces cerevisiae*, *Lysinibacillus sphaericus* CBAM5, and *Cunninghamella elegans* [24]–[26]. *Pseudomonas fluorescens* and *P. aeruginosa* can utilize Fe²⁺, Zn²⁺, Pb²⁺, Mn²⁺, and Cu²⁺ [17]. *Lysinibacillus sphaericus* is effective against cobalt, copper, chromium, and lead [26]. Additionally, *Aspergillus versicolor*, *A. fumigatus*, *Paecilomyces* sp., *Trichoderma* sp., *Microsporum* sp., and *Cladosporium* sp. are used for cadmium bioremediation [27], while *Microbacterium profundis* is employed for iron (Fe) [28]. Microorganisms have been effectively involved in dye bioremediation, demonstrating their capability to degrade various industrial colored-pollutants. *B. subtilis* strains NAP1, NAP2, and NAP4 have been utilized to remediate oil-based paints [29]. *Myrothecium roridum* IM 6482, *Pycnoporus sanguineus*, *Phanerochaete chrysosporium*, *Trametes trogii*, *Nectriella pironii*, and *Aspergillus tamarii* are employed for their ability to degrade industrial dyes [30],[31]. *Micrococcus luteus*, *Listeria denitrificans*, and *Nocardia atlantica* have shown efficacy in treating textile azo dyes [32],[33]. Additionally, *Bacillus* spp., *P. aeruginosa*, and *Bacillus pumilus* are involved in the bioremediation of textile dyes such as Remazol Black B, sulfonated di-azo dye, and reactive red HE8B, as well as RNB dye [34],[35]. Furthermore, *Bacillus firmus*, *Bacillus macerans*, *Staphylococcus aureus*, and *Klebsiella oxytoca* have been used for the bioremediation of vat dyes and textile effluents [21],[36],[37].

Many microorganisms can degrade petroleum-based hydrocarbons in contaminated soil, including bacteria, fungi, and microalgae [38]–[40]. Notable examples include *Streptomyces* sp., which can degrade n-alkanes (C6–C30), aromatic compounds, and polycyclic aromatic hydrocarbons (PAHs) up to 95% within 7 days [40]. *Pseudomonas aeruginosa* NCIM 5514 can degrade (up to 60.63%) crude oil (C8–C36) within 60 days [39]. *Bacillus subtilis* DM2 is capable of degrading (up to 53.92%) petroleum hydrocarbons (C12, C14, C15) within 4.7 days [41], while the BL-27 strain can degrade crude oil up to 65% within 5 days [42]. *Aspergillus fumigatus* Shu2 demonstrates degradation of total petroleum hydrocarbons up to 57% within 16 days [43]. Among microalgae, *Chlorella* sp. and *Dunaliella salina* can degrade diesel oil up to 52.1%–68.7% and 46.99%–60.3%, respectively, within 9 days [23], and *Chlorella vulgaris* can degrade crude oil, targeting both light and heavy compounds up to 94.3% and 88.2%, respectively, within 14 days [38].

Plastic pollution caused by polyethylene terephthalate (PET), polyethylene (PE), low-density polyethylene (LDPE), high-density polyethylene (HDPE), polystyrene (PS), expanded polystyrene (EPS), polyvinylchloride (PVC), or polycarbonate, among others, is one of the most significant threats to the environment due to their non-degradable nature, with particular concern for microplastics, which have been linked to immediate fatalities in aquatic organisms upon ingestion [44],[45]. Conventional physical and chemical methods for degrading plastic waste have been criticized for exacerbating environmental problems [46]. Due to that, microorganisms have emerged as a sustainable solution for the biological degradation (biodegradation) of plastics [22]. These microorganisms break down plastics by secreting metabolites, like polyhydroxyalkanoate depolymerases, facilitating an efficient, cost-effective, environmentally friendly, and sustainable degradation process. For instance, *Pseudomonas fluorescens* can degrade PE in 270 days, achieving a biodegradation efficiency of 18.0% [22]. *Bacillus siamensis* degrades (with an 8.46% biodegradation efficiency) LDPE in 90 days [47]. *Aspergillus flavus* achieves 5.5% of biodegradation efficiency for HDPE in 100 days [20]. A*spergillus nomius* RH06 degrades 6.63% of LDPE in 45 days [48], and *Bacillus cereus* strain A5 degrades 35.72% of LDPE in 112 days [49]. *Aspergillus oryzae* strain A5 also degrades 36.4% of LDPE in 112 days [49], while *Klebsiella pneumoniae* CH001 achieved an 18.4% biodegradation of HDPE in 60 days [50].

As observed, microorganisms have been proven to be efficient solutions for bioremediation. Currently, the scientific community is focused on understanding the essential intramolecular mechanisms (enzymatic processes, metabolic pathways, redox reactions, among others) to optimize remediation by microbial degradation processes. Exploring the potential of microbial consortia for enhanced bioremediation efficiency is also a priority [51],[52]. Furthermore, research efforts should also address challenges such as the scaling up process for bioremediation, envisioning practical applications, and assessing the impacts of microbial bioremediation strategies through comprehensive sustainable assessments, including life cycle and techno-economic evaluations to develop more effective and sustainable solutions for mitigating environmental pollution using microorganisms.

## 3. Engineering microorganisms

The idea of employing genetically engineered microorganisms (GEMs) for bioremediation emerged from the middle and end of the 1980s and opened new perspectives in the manipulation of microorganisms to be applied in in situ bioremediation.

In the development of GEMs, several key procedures are typically undertaken:

1. Modification of protein expression and regulation: This involves altering the expression levels of enzymes involved in degradation pathways to optimize their activity.
2. Pathway engineering and regulation: New metabolic pathways can be introduced, or existing pathways can be modified to enhance degradation capabilities.
3. Bioprocess optimization, monitoring, and control: Techniques for optimizing bioremediation processes, as well as monitoring and controlling the activities of GEMs in the environment, are crucial for effective bioremediation.

Essential genes in bacteria, those necessary for their survival and basic functions, are typically found on their single chromosome. However, genes encoding enzymes needed for the breakdown (catabolism) of specific or unusual substrates might be located on plasmids [53].

The ability to create recombinant microorganisms for bioremediation of toxic compounds in the environment has become a key goal in the pursuit of biosafety [54]. By manipulating the genetic makeup of microorganisms, scientists can enhance their ability to break down pollutants, thus offering a potentially more efficient and sustainable solution for environmental cleanup. One key aspect of this approach involves identifying and understanding the genes to achieve biodegradation of prominent pollutants within microorganisms. These genes, often located on large conjugative plasmids, encode enzymes and proteins that enable the breakdown of hydrocarbon molecules. Some recent examples are listed in Table 1. Through genetic engineering, researchers can modify these genes or introduce them into other microbial strains to enhance their degradation capabilities. However, despite the biochemical design of pathways and the introduction of specific genes, GEMs may not always perform as expected in real-world conditions. Factors such as environmental variability, competition with native microorganisms, and genetic stability can affect the efficiency and reliability of GEMs in bioremediation efforts. Additionally, the selection of plasmids and donor bacteria in wastewater treatment processes is a crucial consideration. Operators must carefully assess the compatibility and effectiveness of these components to ensure optimal performance and minimize potential risks associated with the release of genetically modified organisms into the environment.

While the potential risks and uncertainties associated with GEMs must be carefully evaluated and managed, their ability to adapt and thrive in diverse environmental conditions can make them valuable tools as a first barrier against environmental pollution. Continued investments in research and development are essential for advancing bioremediation technologies to maximize their effectiveness as sustainable solutions for environmental remediation, minimizing potential risks for ecosystems and human health. Collaborations between scientists, environmental engineers, and regulatory authorities are essential to ensure the safe and effective deployment of genetically engineered microorganisms for pollution control and remediation [54]–[57].

Conventional genetic engineering has always played a crucial role in its prerogative of introducing genes into acceptor organisms. The reconfiguration of a complete metabolic pathway requires the introduction of many gene clusters [58]. Among the most recent genetic engineering techniques, some deserve mention, such as CRISPR and TALEN. These gene editing tools have been studied for some time. In both cases, we are talking about molecular scissors. As the name suggests, both can be considered organic cut and sewing kits. CRISPR works thanks to the presence of three elements: a Cas9 enzyme, a guide RNA, and a DNA sequence that replaces the cut part. What CRISPR does is provide a DNA sequence that can replace the cut part, avoiding all those mechanisms that the cell would naturally implement to repair the damage. So, in practice, CRISPR allows to removal of a “sick” gene by correcting it with a healthy one, supplied directly to the cell. Like CRISPR, TALEN is another genetic engineering tool that acts like molecular scissors. TALEN can bind and recognize a specific DNA sequence. These codons scan the DNA in both directions, nucleotide by nucleotide, and when they have found the complementary sequence, the cut is made. A more effective system, but also much more expensive and more difficult to implement, is the Zinc-finger nucleases (ZFNs), which uses natural proteins that bind the DNA in a sequence-specific manner, allowing the nuclease to cut a specific location [59]. All these are essential tools in the field of genome editing. They allow precise alterations to the genomes of higher organisms by leveraging endogenous DNA repair mechanisms.

It is widely known that environmental contaminations can occur directly or indirectly. Direct contamination happens through contact with sick individuals. Indirect contamination occurs when contaminants spread via vehicles like water, air, soil, food, and other animals. Additional elements of environmental contamination include the combustion of fossil fuels, waste dumping, mismanagement of waste, and activities like extraction and deforestation. The specific genetic modifications intended for the creation of GEMs are aimed to improve, and sometimes introduce ex novo, the ability to degrade polluting substances. They can be adapted to specific target contaminants more efficiently than naturally occurring microorganisms. The use of genetically modified microorganisms in polluted waters and soils plays an essential role in the bioremediation of heavy metals. These contaminants are persistent in the environment since they cannot be degraded or destroyed. When ingested through food, drinking water, or air, they can accumulate in living cells, potentially leading to toxicity [60]. They are responsible for neurological disorders, Parkinson's, Alzheimer's, depression, schizophrenia, cancer, poor nutrition, lack of hormone balance, obesity, abortion, and respiratory and cardiovascular diseases [61].

**Table 1.** Engineered bacteria and their function in bioremediation.

Pollutants	Bacteria	Function	References
Arsenic	*Sphingomonas desiccabilis* and *Bacillus idriensis*	As-removal by bio-volatilization	[57]
	*Escherichia coli*	Reducing As (III) and As (V) accumulation by chelating Metallothionein (fMT)	[62]
	*Escherichia coli*	Reducing As (III) by S-adenosylmethionine	[63]
	*Escherichia coli*	As-removal by metalloregulatory protein ArsR	[64]
	*Rhizobium leguminosarum* bv *trifolii* strain *R3*	As-methylation by As (III) S-adenosylmethionine(SAM)methyltransferase	[65]
Cadmium	*Bacillus subtilis* BR15*1 (pTOO24)*	Luminescent sensors	[66]
	*Caulobacter crescentus* JS4022/p723–6H	Cd (II) sequestration by Hexa-histidine (6His) peptide	[67]
	*Pseudomonas fluorescens OS8*	Luminescent sensors	[68]
	*Escherichia coli*	Cd-removal by phytochelatin synthase	[69]
	*Escherichia coli* JM109	Cd-removal by metallothionein MT	[70]
	*Pseudomonas putida* 06909	Cd-binding by peptide EC20	[71]
	*Mesorhizobium huakuii B3*	Cd-removal by phytochelatin synthase (PCS)	[72]
	*Ralstonia eutropha* MTB	Adsorbing Cd (II) by chimeric MTb	[73]
Nickel	*Escherichia coli*	Overexpression of Serin acetyltransferase	[74]
	*Pseudomonas fluorescens* 4F39	Nickel transport system	[75]
	*Kokuria flava, Desulfovibrio desulfuricans (immobilized on zeolite) Flavobacterium sp., Bacillus firmus, Micrococcus sp*.	Sulfate-reducing bacteria (SRB)-immobilized zeolite carriers	[76]
	*Escherichia coli* SE5000	Nickel transport system and metallothionein	[77]
Copper, lead, chromium	*Kokuria flava*, *Desulfovibrio desulfuricans Flavobacterium* sp., *Bacillus firmus*, *Micrococcus* sp.	Sulfate-reducing bacteria (SRB)-immobilized zeolite carriers	[77]
	*Pseudomonas fluorescens* OS8	Luminescent sensors	[68]
	*Escherichia coli* Jm109	Cloning the human metallothioneins MT2A and MT3 into *Escherichia coli* Jm109 to assess the removal and reduction of hexavalent chromium (CrVI)	[78]
Pesticides	*Escherichia coli* (GEB)	Enzyme possessing the degradability to organochloride pesticides, organophosphorus pesticides, carbamates, and pyrethroid insecticides.	[79]
	*Sphingomonas* sp. strain HJY	Interaction of endophyte plant capable of degrading chlorpyrifos (CP)	[80]
	*Rhodopseudomonas palustris* PSB-S	Pyrethroid detoxification by Est3385 protein	[81]
Xenobiotics	*Burkholderia cepacia* L.S.2.4	Phytoremediation of volatile organic xenobiotics by inserting the pTOM toluene-degradation plasmid	[82]
	*Acinetobacter* ADPWH_lux	Naphthalene degrading	[83]
	*Pseudomonas putida* MC4 and *Pseudomonas putida* MC4-5222	TCP-degrading strain by haloalkane dehalogenase from *P. putida* MC4	[84]
	*Escherichia coli*	Cloning of laccase	[85]
	*Cyanobacterium synechocystis* sp. PCC6803	Biphenyl degradation pathway	[86]
	*Rhizosphere bacteria*	Horizontal transfer C230 genes for phenol degradation in soil	[87]
	*Pseudomonas putida* PaW85	Biodegradation of oil-polluted soil	[88]
Agro-industrial waste	*Paracoccus aminophilus* CRT1 and *Paracoccus kondratievae* CRT2	Carotenoid-producing strains of *Paracoccus* carrying a new plasmid pCRT01	[89]
	*Escherichia coli*	Overexpression of Capsanthin/Capsorubin synthase from *Capsicum annuum*	[90]
	*Rhodosporidium toruloides*	Genes for lipid and carotenoid production expression	[91]
	*Saccharomyces cerevisiae*	Expression of cassette carrying a cellulase gene from *A. gigas* Spix	[92]
	*Saccharomyces cerevisiae*	Improvement of lignocellulosic biomass conversion into ethanol	[93]

## 4. Factors affecting microbial bioremediation

Bioremediation is a process that employs microorganisms, primarily bacteria or fungi, to facilitate the breakdown or transformation of harmful pollutants into less dangerous forms. In practical terms, the metabolic processes of microorganisms are leveraged for this purpose. These organisms serve as natural catalysts, aiding reactions that deactivate contaminants. However, for bioremediation to be effective, the environment must provide suitable conditions for microbial life. Microorganisms can only act on pollutants if they have access to various substances that provide the necessary nutrients and energy for their growth. In some instances, the natural conditions of a contaminated site can naturally support bioremediation without human intervention, a phenomenon known as intrinsic bioremediation [94].

The microorganisms' metabolism and the characteristics of contaminants they have to interact with influence the nature of the interaction. However, the specific outcome of this interaction is contingent upon the environmental conditions prevailing at the location where it occurs. In order to ensure the success of a bioremediation process, specific criteria must be addressed, encompassing the microbiological, chemical, and environmental conditions (such as soil type, temperature, pH, oxygen levels, and nutrient availability) of the site in question.

The decomposition of organic compounds is largely affected by living organisms. This is facilitated by several processes, such as microbial competition for carbon resources, antagonistic interactions between microorganisms, and predation by protozoa and bacteriophages. The degradation rate of contaminants depends on their concentration and the presence of organisms that can metabolize them. Additionally, the production of specific enzymes influences the degradation rate. Other important factors include mutation, gene transfer, enzyme activity, and the interactions between various microbial communities [94]–[96].

As will be detailed in the following sections, maintaining the appropriate oxygen levels in the soil is essential for enhancing bioremediation efficiency by either promoting or inhibiting specific microbial communities. However, the use of hydrogen peroxide is constrained due to its toxicity to microorganisms at high concentrations (above 100 ppm, or 1,000 ppm with proper acclimatization). Additionally, hydrogen peroxide quickly breaks down into water and oxygen in the presence of certain soil components. Anaerobic conditions can be employed to degrade highly chlorinated contaminants, although this process is inherently slow. Subsequent aerobic treatment can then be used to complete the biodegradation of partially dechlorinated compounds and other pollutants [96].

Pollutants need to be bioavailable to be suitable for biological degradation. This bioavailability is contingent upon both the physical form of the pollutant and the likelihood of effective interaction between microorganisms and pollutants. A high interface between microorganisms and pollutants will result in a better interaction among them [97].

Polar contaminants that readily dissolve in water are inherently more accessible for biological processes. However, enhancing the interaction between microorganisms and hydrophobic contaminants may necessitate the use of surface-active agents. To understand how accessible a chemical compound to microorganisms is and its potential for biodegradation, it is necessary to know how it moves and if it is distributed in different chemical forms (dissolved, adsorbed, and volatile) in the environment. Bioavailability encompasses the combined impacts of various physical and chemical factors that ultimately influence the microbial utilization of a compound, and consequently, its potential for biodegradation [98].

Regulation of nutrient levels is essential to promote microbial growth and improve biodegradation efficiency. Optimizing essential nutrients such as nitrogen and phosphorus can increase biodegradation rates by adjusting the C:N:P ratio of bacteria [99]. Microorganisms depend on carbon, nitrogen, and phosphorus for their survival and metabolic activities, but low concentrations of these elements limit, for example, the degradation of hydrocarbons. An additional benefit of adding appropriate nutrients is the ability to increase microbial metabolic activity, especially in cold environments. Indeed, the availability of nutrients is a key factor limiting biodegradation in marine conditions [100]. In a similar manner to the nutritional requirements of other organisms, oil-consuming microorganisms also depend on nutrients for optimal growth and development. While these nutrients are present in the natural environment, they are typically present in low concentrations [101]–[103].

Physical and chemical factors including redox potential (Eh), pH, ionic strength, solubility, the presence or absence of electron acceptors and donors, and temperature, are intricately linked and crucial for the success of bioremediation. Redox potential (Eh), ionic strength, and solubility are of paramount importance in the context of bioremediation. The redox potential affects the availability of electron donors and acceptors, with different conditions favoring specific microbial processes for contaminant degradation. Ionic strength influences contaminant mobility and microbial activity. It must be optimized for effective bioremediation. Solubility determines contaminant bioavailability, with strategies such as surfactant-enhanced solubilization used to improve microbial access. A comprehensive understanding and effective management of these factors is critical for efficient contaminant degradation in polluted environments [99],[101],[103].

Additionally, pH plays an important role in bioremediation efficiency. For instance, biosorption, an initial step in the removal of toxic metals by microorganisms, is heavily influenced by pH, which affects the isoelectric point of a solution, determining the net negative charge on microbial cell surfaces or changing the ionic state of ligands like carboxyl residues, phosphoryl residues, or S-H and amino acid groups. Additionally, pH levels impact the solubility of metal ions, which increases as the medium's pH decreases, thereby affecting their uptake by microbial cells [104].

Temperature is a critical factor for the survival of microorganisms and the degradation of hydrocarbons. In cold environments such as the Arctic, natural oil degradation processes are slow, hampering the ability of organisms to clean up oil spills. The subzero temperatures in these areas can cause microbial cells' transport channels to close or even freeze their entire cytoplasm, making most oil-degrading organisms metabolically inactive. The biodegradation of organic compounds depends on the optimal temperature requirements of the compounds involved and the metabolic turnover rates of the degrading organisms. In addition, the degradation of specific compounds is influenced by temperature [104].

Furthermore, alpine regions exemplify another environment with low temperatures that support a diverse array of microorganisms. These microorganisms play a crucial ecological role in Alpine ecosystems, participating in essential processes such as nutrient cycling. Over the past 20 years, many studies have demonstrated that Alpine microorganisms can efficiently degrade various hydrocarbons, including phenol, phenolic compounds, and petroleum hydrocarbons. Moreover, the potential for low-temperature bioremediation of European Alpine soils by enhancing the degradation capabilities of native microorganisms has also been validated [105]–[107].

## 5. Main bioremediation approaches

Bioremediation frequently occurs in complex, multiphase, and heterogeneous environments, such as soils where contaminants are linked with soil particles, dissolved liquids, and gases. Due to this complexity, successful bioremediation necessitates a multidisciplinary approach, integrating expertise from microbiology, engineering, ecology, geology, and chemistry.

**Figure 1.**
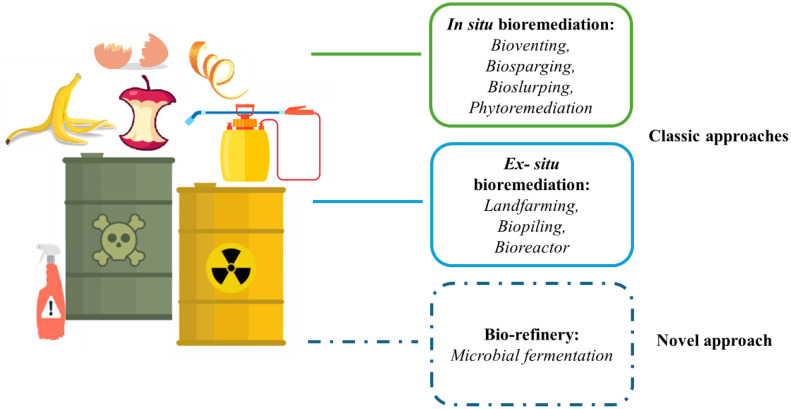
Bioremediation approaches.

Bioremediation can be managed through two distinct approaches, in situ and ex situ, depending on the specific conditions and needs of the situation. The former approach includes techniques such as bioventing, biosparging, and phytoremediation along with physical, chemical, and thermal processes. It circumvents the need to transport contaminants and minimizes the environmental impact. It is considered a practical and sustainable method to selectively destroy organic pollutants without harming flora and fauna and can be applied to pollutants present in low but environmentally significant concentrations [108]. Moreover, as stated beyond, a novel approach to bioremediation can be addressed by biomass waste biorefinery (Figure 1).

### 5.1. In situ environment bioremediation

#### 5.1.1. Bioventing, biosparging, and bioslurping

Bioventing involves enhancing the activity of native microorganisms in the subsoil by introducing atmospheric air (or oxygen) into the unsaturated zone of the subsoil via extraction or injection wells and adding nutrients as necessary. In general, compounds that can be degraded aerobically are treatable by bioventing. This method is particularly employed for removing medium-weight petroleum compounds, such as gasoline, kerosene, fuel oil, and lubricants, as lighter compounds (such as petrol) tend to volatilize more readily and are removed more expeditiously by another technique such as soil vapor extraction (SVE). The bioventing technique employs a vacuum soil vapor extraction system, which creates a pressure gradient in the soil. Atmospheric oxygen then flows into the subsoil, initiating a contaminants aerobic decomposition process. In many cases, it is necessary to add supplements such as nitrogen salts, which can be spread as a nutrient solution on the soil or injected into the soil at the contaminated area [109]. Biosparging is a technique that involves the injection of atmospheric air into the aquifer. It can be employed in both saturated and unsaturated soil areas. This method is particularly effective for reducing the concentration of dissolved oil components in groundwater, capillary fringes, and absorbed into soil below the water table [8]. The main goal of this technique is to reduce energy consumption. The air injected into the aquifer creates small channels allowing the air to move toward the unsaturated zone of the soil [110],[111]. To form the numerous branches needed in these channels, the air must be injected into the soil in pulses. The biosparging process facilitates the transport of volatile contaminants to the unsaturated zone, thus necessitating the concurrent use of soil vapor extraction to collect and treat volatile vapors at the surface. To ensure the effectiveness of biosparging, it is necessary to consider several factors. First, it is important to evaluate soil permeability and contaminant biodegradability. Petroleum compounds typically biodegrade in the presence of sufficient oxygen; however, a thorough analysis of soil layers is necessary to ascertain this. The presence of fine-grained materials and soil heterogeneity can hinder the process by restricting airflow. Additionally, oxygen-induced iron precipitation can decrease permeability. It is also essential to assess bacterial growth rates, which depend on temperature and pH and vary with different microorganisms. Laboratory tests on soil samples are critical, as a minimum bacterial population of 10³ CFU/g is needed for biodegradation. If this threshold is not addressed, introducing microbial cultures and nutrients can enhance the process and support cell growth [112]. Feola [112] reported a case study of a site contaminated by polycyclic aromatic hydrocarbons (PAHs) and petroleum hydrocarbons (TPHs), where high abatement efficiencies were achieved in both soil and groundwater using biosparging.

Bioslurping is a technique that is also employed to treat free product phases that are floating on the surface of groundwater. This technique utilizes a vacuum to extract soil vapor, water, and free products from the subsurface. Once extracted, these components are separated and treated. The economic advantage of this technique lies in the fact that only a small amount of groundwater and soil vapor is pumped out at a time, allowing the use of a smaller treatment plant [5].

#### 5.1.2. Phytoremediation

Phytoremediation is an *in situ* technique that uses plants to remediate contaminated soils. Deep-rooted trees, grasses, legumes, and aquatic plants are widely used in phytoremediation. The plants used should be as disease- and insect-resistant as possible [113]. Although phytoremediation can be carried out by most plants already present in a polluted site, in these environments, native plants may be bioaugmented by natural or anthropogenic plants, or a combination of both, as well as by endogenous or exogenous plant growth–promoting rhizobacteria (PGPR). PGPR have been reported to be able to increase the biomass production and tolerance of plants to soil pollutants as a consequence of the utilization of different compounds (sugars, fatty acids, growth factors, amino acids, etc.) released by plants' roots by the symbiotic microflora [1],[8].

### 5.2. Ex situ environment bioremediation

#### 5.2.1. Landfarming, biopiling, and bioreactor

Ex situ remediation encompasses a range of methods, including landfarming, biopiling, and treatment using bioreactors, in addition to thermal, chemical, and physical processes. Although it is considered a more comprehensive reclamation technique, due to the associated costs including excavation and soil transport, in situ reclamation techniques are preferred.

Landfarming is a process whereby contaminated soil is excavated and mechanically screened. The soil is then spread in layers no thicker than 0.5 meters. A synthetic barrier, cement or clay, is then applied to the soil, which is subsequently covered with a layer of soil. Oxygen is then introduced, and the soil is mixed by ploughing, harrowing, or milling. As required, nutrients can be added and moisture can be promoted to enhance the reclamation process, while the soil pH is maintained at a value close to 7.0 using crushed limestone or agricultural lime [112].

Biopiling is the most widely used bioremediation technique for the remediation of contaminated soils. The procedure involves the collection of contaminated soils and their subsequent treatment in structures called piles. In these piles, conditions such as oxygen concentration, soil moisture, nutrient concentration, and pH are controlled to optimize the growth and activity of the indigenous microbiota [114]. The intervention comprises the excavation of the contaminated soil, followed by the mixing of the soil with soil improvers and their transfer to a treatment area. The excavated soil is arranged in overlapping layers, alternating between perforated pipes for the distribution of air and nutrient solutions into the contaminated material and pipes for the extraction of air from the pile. This remediation technique stimulates the growth and multiplication of aerobic bacteria using oxygen by circulating air in the soil through pipes using extraction and injection techniques [5],[115]. Nutrients such as mineral fertilizers or micro-organisms such as fungi, bacteria, or enzymes can also be added in combination [116].

A series of biological reactions takes place within a bioreactor, transforming raw materials into specific products. The use of a bioreactor to treat contaminated soil has numerous advantages over other techniques, such as obtaining a specific pH or the desired temperature, degree of aeration, and the right inoculum concentration. These advantages reduce time and optimize the bioremediation process [5].

### 5.3. Biomass waste biorefinery

For a long time, the concept of biorefinery has been closely linked to energy production by biomass implementation for biofuel production and for allowing GHG emissions reduction [117]. However, the term biorefinery could be extended to other sectors at the industrial scale if products that can only be obtained from agro-industrial and foodstuffs are included [118]. Considering the environmental pollution caused by the landfill or burning of these biomasses, and the possibility of their valorization via biological treatments, it is logical to apply the concept of integrated biorefineries as the facilities that usually reduce waste management and wastewater treatment, considering different raw materials, transforming the biomass into human and animal food products, biomaterials, biofuel, and other value-added products, reducing the environmental pollution [118]. In this sense, the municipal, agro-industrial, and food waste biorefinery carried out by employing microorganisms can be considered a bioremediation technique, based on the bioeconomy principles.

## 6. Agro-industrial waste bioremediation for circular bioeconomy

The agricultural crop processing, post-harvesting operations, and industrial processes by-products generate massive volumes of residues, which act as pollutants to the environment if disposed of and released untreated, affecting humans and other living beings [119]. Agro-industrial residues left unutilized contribute to waste generation, which is expected to reach 3.40 billion metric tons by 2050 [120],[121].

The chemical composition of the agro-industrial waste varies and depends on the processed starting material. Overall, they are rich in suspended solids, nitrogen, phosphorus, organic substances, high biological oxygen demand (BOD), and chemical oxygen demand (COD), which can cause environmental pollution [122].

The conventional methods of waste management via landfilling, open dumping, burning, or incineration of waste cause significant environmental problems with respect to air pollution and groundwater contamination [123],[124]. Henceforth, the focus has shifted to waste valorization, which provides the dual benefit of sustainable production of value-added products and waste management [124], essential prerequisites for sustainable development, contributing to the attainment of the global sustainability goals (SDGs 12 and 13).

Most technological agro-industrial waste valorization procedures are focused on the generation of biofuels or bioenergy [125],[126]. They are currently being investigated as promising substrates for the production of several value-added products, including enzymes, platform chemicals, bioactive molecules, pigments, and so forth [127]–[130]. Moreover, these agro-industrial residues have been found to be suitable as nutrient support for microorganisms' growth in order to produce value-added products such as single-cell protein (SCP) and single-cell oil (SCO) by both submerged and solid-state fermentation. This approach not only combats environmental problems but also provides a sustainable and cost-effective framework for developing a circular bioeconomy [119],[131]–[134].

**Figure 2.**
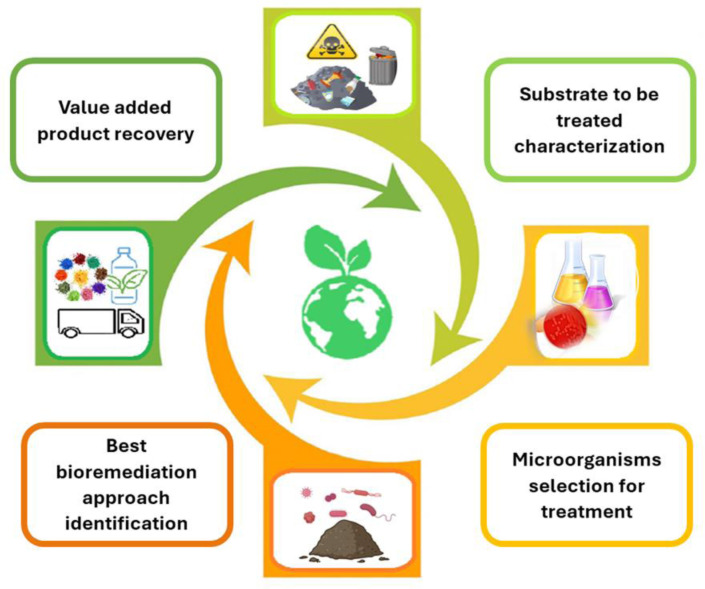
Bioremediation bioeconomy-based outline.

The valorization of these wastes will help to reduce environmental pollution and health hazards and, at the same time, develop economical bioprocess. In this regard, it is important to have an extended vision of bioremediation connecting it to agro-industrial waste biorefinery, embracing the updated concept of the process reported by Conteratto et al. [117] as “a physical, chemical, or biological process which purifies, separates, refines, or transforms elements constituting biological assets from the kingdoms Monera, Protista, Plantae, Animalia, or Fungi, originating from the terrestrial or oceanic environment, in bioproducts for final use or that serve as raw material for other bioproducts” (Figure 2).

### 6.1. SCP and SCO production

The sustainable bioremediation approach through microorganisms' utilization of organic pollutants as nitrogen or carbon sources may yield protein- or oil-enriched biomasses, called single-cell protein (SCP) or single-cell oil, respectively (SCO), reducing the organic pollutants from agro-industrial waste [119],[135]–[138] and representing a novel and environmentally benign technology [139].

SCPs are proteins of microbial origin obtained by pure or mixed cultures of bacteria, fungi, yeasts, or microalgae by submerged, semi-solid, or solid-state fermentation that can be used as protein supplements [140]. Their production from agro-industrial waste represents a promising microbial-based technology that has recently gained increased interest due to their potential environmental benefits over traditional protein supplements [141]. SCPs show a massive spectrum of applications, from animal feed supplements to paper processing and novel packaging materials production [142]. Agro-industrial waste represents a well-investigated substrate for SCPs production by several microorganisms. Many fungal genera have been widely employed, including *Saccharomyces*, *Candida*, *Rhodotorula*, *Aspergillus*, *Neurospora*, *Fusarium*; among bacteria, the most appealing are *Lactobacillus*, *Bacillus*, *Methylomonas*, and *Methylococcus*; finally, among microalgae, the most employed are *Spirulina*, *Chlorella*, *Dunaliella*, *Haematococcus*, and *Schizochytrium* [139],[142],[143].

SCOs are produced by oleaginous microorganisms, including yeast, fungi, bacteria, and microalgae, that are able to accumulate lipids to up to more than 20% of their biomass in the form of triglycerides (TGAs) and free fatty acids, showing a high percentage of essential fatty acids [136],[138]. Among the oleaginous microorganisms, the potential candidates for their ability to metabolize low-cost substrates as agro-industrial waste are filamentous fungi belonging to the genera *Mortierella* and *Mucor* [144]. SCOs are largely used both in food and feed industries as well as in the bioenergy sector for biodiesel production [138].

### 6.2. Bio-based energy production

Bio-based energy, such as biofuels, biogas, hydrogen, and electricity, is renewable energy obtained from living organisms using agricultural wastes as fermentation substrates [119]. Bio-based energy production starting from agro-industrial waste will have a positive impact not only on decreasing environmental pollution but also on mitigating greenhouse gas emissions while replacing the implementation of fossil fuels with bioenergy [134].

Biofuel production from agro-industrial waste is one of the most popular alternative energy production pathways [126]. Biodiesel, biogas, bioethanol, and biomethane are the main biofuels produced by several microorganisms such as yeast, bacteria, fungi, and microalgae [145],[146]. Among the biofuels, bioethanol is considered the most sustainable alternative to gasoline, which can be easily obtained by different fermentation methods such as direct fermentation (DF), separate hydrolysis and fermentation (SHF), and simultaneous saccharification and fermentation (SSF) [133] by applying different microorganisms, mainly belonging to *Saccharomyces*, *Schizosaccharomyces*, *Candida*, *Torulopsis*, and *Escherichia coli* [147],[148].

Biogas production has been reported as the most used process for agro-industrial waste bioremediation [1]. This technology is based on the employment of specialized microbial populations, mainly belonging to the phyla *Firmicutes*, *Actinobacteria*, *Bacteroidetes*, *Chloroflexi*, and *Proteobacteria*, that are able to convert this waste to water, methane, and carbon dioxide through anaerobic digestion [149].

In recent years, anaerobic digestion has also been applied for microbial electrolysis cells (MEC) in order to increase the hydrogen yield from organic waste remediation [1],[150], being called microbial remediation cell (MRC) in this case. The MEC technology consists of two electrodes: an anode, where the microorganisms degrade the feed, and a cathode, where hydrogen is produced. The most used microorganisms are obligate anaerobic bacteria belonging to *Shewanella* sp., *Geobacter* sp., *Pseudomonas* sp., *Rhodoferax* sp., *Rhodopseudomonas* sp., classified as hydrolyzing bacteria able to break down the polymers to monomers and electrochemically active bacteria that can also oxidize monomers into electrons and transfer them to the anode [151].

Finally, the employment of different agricultural wastes and wastewater for bioelectricity production in microbial fuel cells (MFCs) is gaining more attraction as a promising and alternative source of renewable energy generation [152],[153]. The conversion of chemical energy into electrical energy in the MFCs majorly depends on the biodegradation efficiency of the solid organic agro-industrial waste by the anodophilic microorganisms, mainly represented by wild-type and engineering *Shewanella oneidensis*, *Comamonas*, and *Acinetobacter* [147],[154].

### 6.3. Value-added products produced by agro-industrial waste bioremediation

Recycling is the most important feature for product design and optimization in a circular bioeconomy. Waste outflows produced by food and sugar processing industries (i.e., plant oils, whey, glycerol, molasses, leftover coffee grounds, fruit wastes, and lignin-rich wastes) might be regarded as a suitable carbon substrate for biopolymers, surfactants, pigments, flavor and aroma compounds, and enzymes [123],[124],[155],[156].

Today, searching for biodegradable “green plastics” obtained by microbial fermentations is one of the most important goals for sustainability. Microbial biopolymers are eco-friendly, 100% perishable, non-toxic, and biocompatible [157]. Those most produced are represented by polyhydroxyalkanoates (PHAs). Several studies have been reported on the implementation of agro-industrial waste for PHAs production by using microorganisms such as *Bacillus megaterium*, Bacillus *cereus*, *Bacillus subtilis*, *Pseudomonas aeruginosa*, *Pseudomonas putida, Halomonas campisalis*, *Pseudomonas fluorescens, Pseudomonas oleovorans, Ralstonia eutropha, Paracoccus sp*. and *Cupriavidus necator* [155],[158]–[161].

Agro-industrial waste can be used as a feedstock for natural pigment-producing microorganisms by applying both submerged and solid-state fermentation techniques [124]. Bacteria, yeast, fungi or microalgae are able to produce a diverse array of pigments (carotenoids, flavins, anthraquinones, violacein, and prodigiosin) as secondary metabolites, which can be used for different industrial applications such as pharmaceutical, food, feed, textile, cosmetics, sensors, and energy [119],[124],[162]–[164].

Carotenoids, prodigiosin, tambjamines, melanins, quinones, and violacein are the most commonly pigments produced by bacteria such as *Gordonia jacobaea*, *Serratia marcescens*, *Chromobacterium* sp., *Erwinia chrysanthemi*, *Corynebacterium insidiosum*, *Vogesella indigofera*, *Chryseobacterium* sp., *Hymenobacter* sp., *Micrococcus*, *Chryseobacterium artocarpi*, *Kocuria* sp., *Pseudomonas* sp., *Dietzia* sp., *Paracoccus* sp., *Bradyrhizobium* sp., *Brevibacterium* sp., *Agrobacterium* sp., *Streptomyces* sp., and G. *jacobaea* [165].

Specifically, the yeasts *Pichia*, *Rhodotorula*, *Xanthophyllomyces*, *Rhodosporidium*, *Sporobolomyces*, and *Sporidiobolus* are also potent producers of various carotenoids and other pigments. The carotenoids most produced by yeasts include β-carotene, torulene, astaxanthin, and canthaxanthin [165]. Fungi belonging to the families *Chlorociboriaceae*, *Monascaceae*, *Sordariaceae*, *Trichocomaceae*, *Chaetomiaceae*, *Nectriaceae*, *Xylariaceae*, *Hypocreaceae*, *Cordycipitaceae*, *Pleosporaceae*, among others, are also reported as prominent pigment producers [166]. Finally, among microalgae, the genera *Arthrospira*, *Chlorella*, Dunaliella, *Mychonasterotundus*, *Haematococcus*, *Nostoc*, *Phormidium*, and *Porphyridium* are reported as the most implemented for carotenoid and phycobiliprotein production by using agro-industrial waste [167],[168].

Overall, although agro-industrial waste bioremediation is a sustainable strategy for minimizing environmental contamination and simultaneous pigment production, it has shown major bottlenecks. One of the major challenges is the limited potential of natural pigment-producing wild-type microorganisms. Therefore, the implementation of genetic, metabolic, and biomolecular strategies may help in developing improved strains for pigment production by using agro-industrial waste [124].

The bioremediation of agro-industrial waste has been widely applied as a low-cost substrate for the microbial generation of biosurfactants, which can be implemented in further bioremediation processes such as the removal of heavy metals and hydrocarbons from soil, the enhancement of phytoremediation, and the improvement of the efficacy of pesticides and biopesticides [169]–[171]; polylactate (PLA), bioplastic materials produced by agro-industrial residue lactic acid fermentation and mainly used for packaging [123]; and aroma and flavor compounds to be implemented as additives in food, cosmetic, and fragrance industries [172]. Finally, many studies have focused on producing enzymes representing high commercial-value products. In this regard, a solution for agricultural waste bioremediation is the implementation of microorganisms producing enzymes with industrial properties [156],[172].

## 7. Bioremediation: green solution with hidden challenges

Bioremediation is a sustainable and environmentally friendly solution for the treatment of environmental pollution caused by anthropological development and waste generated by industrial activities. This technique has gained increasing popularity as a cost-effective and viable alternative to traditional waste management methods [174]. However, despite the numerous benefits associated, several critical issues and challenges emerge during the bioremediation process. One of the primary challenges associated with bioremediation is the effectiveness of this method for specific types of pollutants [175]. While this approach has proven effective for a diverse range of contaminants, not all of them can be completely degraded by microorganisms. Some complex or highly toxic chemical pollutants may exhibit resistance to biological processes, necessitating the implementation of specific conditions or additional treatments to achieve effective neutralization [5],[103]. Moreover, the success of bioremediation is contingent upon the prevailing environmental conditions at the site, including pH, temperature, and oxygen availability. The aforementioned factors can vary considerably between sites, rendering a universal approach impractical and necessitating significant adaptations to ensure treatment efficacy [8].

Another factor to be considered is the length of time required for the process to achieve the desired level of pollutant reduction. There are some critical issues in the application of bioremediation strategies; depending on the condition, many pollutants tend to escape the degrading action, mainly because of microbes' inability to interact or attach them, resulting in an impossibility to convert them into harmless compounds [176]. Bioremediation can take weeks, months, or even years to be completed, which may cause problems for the industries that require rapid waste disposal and site remediation and have an adverse effect on the time and cost management of industrial operations. The success of bioremediation is contingent upon the implementation of a rigorous monitoring and control system. The necessity for continuous monitoring is driven by the need to ascertain the continued viability of the microorganisms and the efficacy of the pollutant's degradation process. This introduces a layer of complexity and potential costs to the process, as monitoring necessitates the deployment of human and technological resources for the regular assessment of site conditions and biological activity. Furthermore, there is a risk that bioremediation may not achieve complete degradation of all hazardous substances, resulting in the formation of residual, potentially harmful intermediates. In the event of incomplete degradation, additional intervention or treatment may be necessary, which would result in increased overall costs and complexity [177].

The ecological impact of bioremediation is another critical factor that must be considered. The introduction of non-native microorganisms or large quantities of naturally occurring organisms can disturb local ecosystems. Therefore, the ecological impact of such interventions must be carefully evaluated and managed to avoid negative effects on biodiversity and ecosystem health. Furthermore, the metabolic activities of microorganisms used in bioremediation may sometimes produce secondary pollutants, which may require further treatment.

Some soil contaminants, recalcitrant to microbial action, could enter the food chain through the consumption of plants by animals [176]. The production of potentially harmful by-products must be carefully monitored and managed to avoid further environmental contamination [178].

The technical and logistical challenges inherent to bioremediation represent an additional obstacle to overcome. The design and implementation of a bioremediation strategy can be a complex process that requires the input of specialists with a specific skill set. This may entail pilot studies, feasibility assessments, and bespoke solutions tailored to specific waste types and site conditions. Technical complexity can act as a barrier to the widespread adoption of bioremediation, particularly for small and medium-sized enterprises with limited resources. While bioremediation is typically cost-effective, it still necessitates some infrastructure, expertise, and initial investment for the installation of biological treatment systems [179]. A lack of adequate infrastructures or experience in managing bioremediation may restrict the efficacy and uptake of the process. Additional challenges arise in the form of regulatory compliance and public acceptance. Ensuring that bioremediation practices comply with environmental regulations represents a significant challenge. It is possible that there may be rigorous requirements for monitoring, reporting, and maintaining safe levels of residual contaminants. Bureaucratic and regulatory procedures may potentially impede the implementation of bioremediation projects and increase overall costs [180]. Furthermore, public acceptance of bioremediation projects may vary. Concerns about the safety and effectiveness of using microorganisms to treat hazardous waste may lead to resistance or demands for more information and transparency [181]. Effective communication with the public and building trust are essential for the success of bioremediation projects. The practice of bioremediation represents a significant step toward the development of a more sustainable approach to industrial waste management. New ways are being devised to improve the bioavailability capacity of microbes and add value to the efficiency of bioremediation. Techniques such as waste solubilization by heat injection using hot air, steam or hot water washing, fracturing the underground matrix at high pressure, and adding surfactants are some of the preferred ways to improve existing technologies [176]. However, it is important to recognize that this process involves several complexities and challenges that require careful consideration. It is essential to strike a balance between enthusiasm for green technologies and a critical assessment of their practical applications. As we proceed with bioremediation, it becomes important to develop sustainable strategies for assessing its impact. To achieve the settled objectives, it becomes vital to encourage collaborations between the scientific community, industry, and regulatory authorities. Bioremediation offers an opportunity to innovate in harmony with natural processes for a cleaner and healthier future, in accordance with the 2030 Agenda for Sustainable Development and the Sustainable Development Goals (SDGs) “Goal 9: Build resilient infrastructure, promote inclusive and sustainable industrialization and foster innovation”, “Goal 12: Ensure sustainable consumption and production patterns” and “Goal 13: Take urgent action to combat climate change and its impacts”. Addressing these goals by the implementation of bioremediation techniques, with a particular regard to biorefinery processes, will allow environmental pollution to decrease by functionalizing the circular bioeconomy model. However, to achieve this, it is necessary to demonstrate knowledge, skills, and competences to conduct research and to be prepared for the major challenge represented by the capability to attract stakeholder's investment [182].

Additionally, according to the Bioremediation Market Size, Share & Trends Report 2030 [183], the global bioremediation has been valued at USD 12.38 billion in 2021 and is expected to register a compound annual growth rate (CAGR) of 9.93% from 2022 to 2030. The market is anticipated to witness growth due to rapid industrial development in recent years that has led to widespread contamination of several environmental landscapes, such as oceans, freshwater systems, forests, and agricultural lands. Similarly, mismanagement of plastic waste, crude oil spills, increasing production of greenhouse gases (GHG), and release of chemical pollutants, such as polycyclic aromatic hydrocarbons, bisphenol-A, pyrethroids pesticides, and dioxanes, have led to worsening environmental outcomes and are increasing the demand for bioremediation services.

Because bioremediation is a new technology, a successful bioremediation program requires a multidisciplinary approach, integrating fields such as microbiology, engineering, geology, hydrogeology, soil science and project management [184]. Only through a deep understanding of the potential and limitations of this technology will it be possible to make the most of it and ensure that it contributes to a cleaner and healthier environment for future generations.

## 8. Conclusions

The increase in human population, waste generation, fossil reserves depletion, and land degradation justify the global interest to identify the most promising techniques for reducing environmental pollution, namely by applying a bioeconomy implementation. In this context, the line between bioremediation and biorefinery is very thin; the possibility of obtaining value-added products represents an important goal both from an environmental perspective, reducing pollution, and an economic point of view, due to the simultaneous production of market-value products. Thus, a waste bioremediation-circular bioeconomy approach may ensure a safe environment.

Nevertheless, it is important to consider that, for exporting these innovative and ecofriendly bioprocesses to an industrial scale, many more efforts are required from the scientific community, as well as the implementation of supportive regulatory policies.

This study highlights the microorganism's role in waste/pollution remediation and their significant contribution in managing waste, promoting sustainability, and addressing the transition to a circular bioeconomy. Nevertheless, more investigations are needed to better understand benefits and limitations in this field. The concept of bioremediation will be successfully addressed only through the implementation of optimized technologies and processes resulting from the evaluation of all techno-economic limits.

## Use of AI tools declaration

The authors declare they have not used Artificial Intelligence (AI) tools in the creation of this article.
